# 2944. Comparative Effectiveness and Safety of 5-7 days versus 10 days of Antibiotics for Acute Bacterial Sinusitis in Children

**DOI:** 10.1093/ofid/ofad500.183

**Published:** 2023-11-27

**Authors:** Timothy J Savage, Matthew Kronman, Sushama Kattinakere Sreedhara, Krista F Huybrechts

**Affiliations:** Boston Children's Hospital / Brigham and Women's Hospital, Boston, MA; Seattle Children's Hospital / University of Washington, Seattle, WA; Brigham and Women's Hospital, Boston, Massachusetts; Brigham and Women's Hospital, Boston, Massachusetts

## Abstract

**Background:**

Acute bacterial sinusitis accounts for 5 million pediatric antibiotic prescriptions in the United States each year, with 65% amoxicillin or amoxicillin-clavulanate. More than 90% of pediatric patients are prescribed 10+ days of antibiotics. No study has compared shorter to longer treatment. This study compares 5-7 days versus 10 days of treatment in children and adolescents.

**Methods:**

This cohort study included patients < 18 years with an outpatient encounter for acute sinusitis and a same-day dispensation of either a 5–7 day supply or a 10 day supply of amoxicillin or amoxicillin-clavulanate between January 1, 2017 and December 31, 2020 in the MarketScan Commercial Claims and Encounters database. Treatment failure, defined as a new antibiotic dispensation, an ED or inpatient encounter for acute sinusitis, or an inpatient complication of acute sinusitis, was assessed in the first 5, 10, and 30 days after cohort entry. Adverse and control outcomes were captured. We used Cox proportional hazards regression with propensity score (PS) overlap weights for confounding control.

**Results:**

The initial cohort included 254,629 patients, among which 19,627 patients were prescribed a 5-7-day treatment (exposure) and 235,002 patients were prescribed a 10-day treatment (referent). After PS overlap weighting, groups were balanced on all measured covariates (Table 1). The rate of 30-day treatment failure was 167.77 per 1,000 person-years (95% CI, 155.94, 180.49) among patients dispensed 5-7 days of treatment and 150.19 per 1,000 person-years (95% CI, 145.54, 154.99) among patients dispensed 10 days of treatment (hazard ratio 1.12 [95% CI, 1.03,1.21]) (Table 2). Adverse events were rare, and there was no difference between groups in a negative control outcome (Table 3).

**Table 1:**
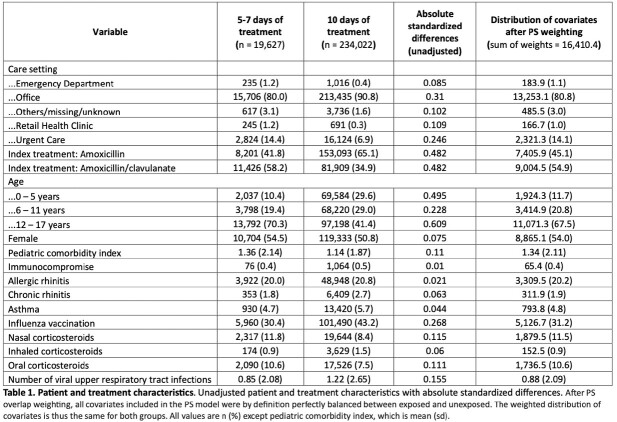
Patient and treatment characteristics

**Table 2:**
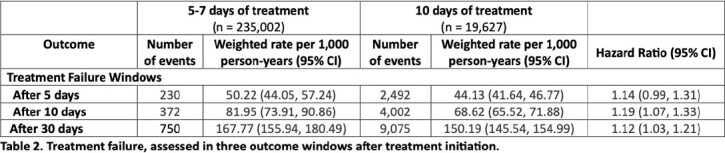
Treatment Failure

**Table 3:**
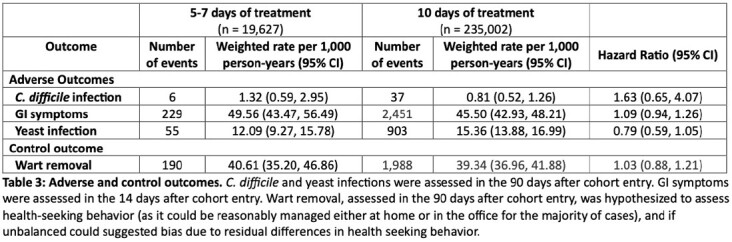
Adverse and control outcomes

**Conclusion:**

The absolute risk of treatment failure was very low. Children and adolescents dispensed 5-7 days of antibiotic treatment had a slightly higher rate of treatment failure compared to 10 days of antibiotic treatment. Adverse effects were rare, with a potentially lower rate of yeast infections following shorter treatment. The marginal benefit and increased risk of side effects with longer treatment indicates that shorter treatment of acute bacterial sinusitis, 5-7 days, may be a reasonable initial approach in some populations.

**Disclosures:**

**Timothy J. Savage, MD, MPH, MSc**, UCB: Contract to Brigham and Women's Hospital **Krista F. Huybrechts, PhD, MS**, Takeda: Grant/Research Support|UCB: Grant/Research Support

